# Dendritic Cells Loaded with Pancreatic Cancer Stem Cells (CSCs) Lysates Induce Antitumor Immune Killing Effect In Vitro

**DOI:** 10.1371/journal.pone.0114581

**Published:** 2014-12-18

**Authors:** Tao Yin, Pengfei Shi, Shanmiao Gou, Qiang Shen, Chunyou Wang

**Affiliations:** 1 Pancreatic Disease Institute, Department of General Surgery, Union Hospital, Tongji Medical College, Huazhong University of Science and Technology, 1277 Jiefang Avenue, Wuhan, Hubei, P. R. China; 2 Department of Clinical Cancer Prevention, The University of Texas MD Anderson Cancer Center, Houston, TX, United States of America; Indiana University School of Medicine, United States of America

## Abstract

According to the cancer stem cells (CSCs) theory, malignant tumors may be heterogeneous in which a small population of CSCs drive the progression of cancer. Because of their intrinsic abilities, CSCs may survive a variety of treatments and then lead to therapeutic resistance and cancer recurrence. Pancreatic CSCs have been reported to be responsible for the malignant behaviors of pancreatic cancer, including suppression of immune protection. Thus, development of immune strategies to eradicate pancreatic CSCs may be of great value for the treatment of pancreatic cancer. In this study, we enriched pancreatic CSCs by culturing Panc-1 cells under sphere-forming conditions. Panc-1 CSCs expressed low levels of HLA-ABC and CD86, as measured by flow cytometry analysis. We further found that the Panc-1 CSCs modulate immunity by inhibiting lymphocyte proliferation which is promoted by phytohemagglutinin (PHA) and anti-CD3 monoclonal antibodies. The monocyte derived dendritic cells (DCs) were charged with total lysates generated from Panc-1 CSCs obtained from tumor sphere culturing. After co-culturing with lymphocytes at different ratios, the Panc-1 CSCs lysates modified DC effectively promoted lymphocyte proliferation. The activating efficiency reached 72.4% and 74.7% at the ratios of 1∶10 and 1∶20 with lymphocytes. The activated lymphocytes secreted high levels of INF-γ and IL-2, which are strong antitumor cytokines. Moreover, Panc-1 CSCs lysates modified DC induced significant cytotoxic effects of lymphocytes on Panc-1 CSCs and parental Panc-1 cells, respectively, as shown by lactate dehydrogenase (LDH) assay. Our study demonstrates that the development of CSCs-based vaccine is a promising strategy for treating pancreatic cancer.

## Introduction

Pancreatic cancer is one of the most lethal malignancies of the digestive system, which ranks as the leading cause of cancer-related death in developed countries. In recent years, the incidence of pancreatic cancer and the related death is steadily increasing in developing countries, whereas the prognosis of most pancreatic cancer patients did not improve over the last thirty years. The majority of pancreatic cancer patients lost opportunity for surgical resection, due to the fact of advanced stage of disease at diagnosis, and intrinsic or acquired resistance to chemotherapy or radiotherapy, which is a typical characteristic of pancreatic cancer [1]. Thus, it is urgent to explore new targeted intervention strategies for treating pancreatic cancer.

The cancer stem cells (CSCs) are a subpopulation of tumor cells that possess strong self-renewal and differentiation abilities to drive carcinogenesis and progression of cancer. Recently, the CD44+CD24+ESA+ pancreatic CSCs have been confirmed to possess an increased tumorigenic potential, and are responsible for the malignant behavior of pancreatic cancer [2]. Complete eradicating of these CSCs may provide hope as a novel therapeutic strategy to treat pancreatic cancer. However, anti-apoptotic ability is one of the prominent features for CSCs across multiple tumor types, resulting in ineffective killing in standard chemotherapy and radiotherapy [3]–[4]. The remnant CSCs may thus become the origin of recurrence and the source of treatment failure.

Cancer immunotherapy activates the patient's antitumor immune responses to reject malignant tumor cells. CSCs express certain biomarkers that have distinct antigenicity, which may induce specific immune responses [5]–[6]. CSCs have been shown to be recognized and eliminated by the CD8+ cytolytic T Cells [7]. Moreover, it has been demonstrated that intrinsic immunity against CSCs may dictate cancer progression course [6]. Thus, it is an attractive strategy to induce immune responses against pancreatic CSCs for the treatment of pancreatic cancer. DCs are potent antigen-presenting cells and play a pivotal role in inducing primary immune responses against tumor-associated antigens. A number of strategies have been developed to modify DCs with tumor specific antigens to generate anti-tumor immune responses. Weak tumor antigen-modified DC vaccine can elicit strong anti-tumor immune responses in vitro and in vivo [8]. In this study, we modified DCs with pancreatic CSCs antigen, and investigated the killing effect of immune responses elicited by CSC-DC on pancreatic CSCs in vitro.

## Materials and Methods

### Ethics statement

The use of human subjects was specifically approved by the Clinical Research Ethics Committee of the Union hospital, Tongji Medical College, HUST. The blood samples were obtained from healthy donors. All the volunteers had known and agreed that the blood was going to use for scientific research. All participants signed a written informed consent form before donating the blood.

### Cell culture

The Panc-1 pancreatic cancer cell line was cultured in Dulbecco Modified Eagle Medium supplemented with 10% fetal bovine serum (Sigma Chemical Co., St. Louis, MO), pencillin (100U/ml) and streptomycin (100u/ml) at 37°C incubator with 5% CO_2_. The culture condition for pancreatic cancer cells to form tumor spheres in suspension was described previously [9]. Briefly, the enzymatically dissociated single cells were diluted to a density of 10^3^ cells/mL in sphere-forming medium (SFM). The cells were passaged every 10 to 14 days and replated in the SFM. The spherical clusters of cells grown in this condition were named Panc-1 CSCs. SFM used was DMEM-F12 supplemented with 10 ng/mL fibroblast growth factor-basic (Peprotech), 20 ng/mL epidermal growth factor (Peprotech), 5 ug/mL insulin, 2.75 ug/mL transferrin, 2.5 ng/mL sodium selenite (Sigma), and 0.4% bovine serum albumin (Amresco).

### Preparation of tumor cell lysate

Pancreatic cancer cells (Panc-1 sphere, Panc-1) were incubated with 0.01% EDTA-solution for 5 min. After washing twice in PBS, the cells were resuspended in serum-free medium. The cell suspensions were frozen at −80°C and thawed by four freeze-thaw cycles. After the removal of crude debris, the lysates were centrifuged for 10 min at 300 g gravity. Supernatants were collected and the protein concentrations of the lysates were determined by Bio-Rad assay (Bio-Rad, Munich, Germany).

### Preparation of pancreatic cancer cells lysates modified DC

Peripheral blood mononuclear cells (PBMCs) from two healthy donors were isolated by Ficoll density-gradient centrifugation. The PBMCs were cultured in RPMI 1640 supplied with 10% serum in the culture plates for adherence at 37°C with 5%CO_2_ for two hours. The nonadherent cells were removed and cultured in medium containing 10U/ml IL-2 for further generation of the lymphocyte population. The adherent cells were harvested and cultured in the presence of GM-CSF (Genzyme,100 ng/ml) and IL-4 (Genzyme,100 ng/ml) for 6 days at 37°C, 5%CO2. Then, the cells were incubated with lysates of pancreatic cancer cell (Panc1 sphere and Panc-1) at a concentration of 100 µg for every 2×10^5^ DCs for 24 hrs. Subsequently, DCs were activated with TNF-α (20 ng/ml) for 24 hrs.

### Immunologic phenotypic analysis

Flow cytometry analysis was performed to detect the immune phenotypes of tumor and dendritic cells. Antibodies include anti-HLA-DR-FITC, anti-HLA-ABC-FITC, anti-HLA-DQ-PE, anti-CD80-FITC, anti-CD54-FITC, anti-CD1a-FITC, and anti-CD86-PE,(eBioscience). FITC-IgG and PE-IgG were used as internal controls. For staining, 5×10^5^ cells were plated into the 96 well U plate (Corning,USA)and incubated with 5 µl of each antibody at 4°C for 30 minutes. After washing with PBS, cells were collected at 1000 g gravity for 5 minutes. Then, the cells were resuspended by 300 µl of PBS and subjected to flow cytometry analysis.

### Inhibition of pancreatic cancer cells on lymphocyte proliferation

Panc-1 CSCs were harvested as stimulator cells. After being pretreated with mitomycin C (25 ug/mL), the stimulator cells were cocultured with the 1×10^6^/ml nonadherent lymphocyte (reactors) at a ratio of 1∶10 in the presence of PHA (sigma) or anti-CD3 monoclonal antibodies (OKT3, eBioscience). Then 20 µl 5 ug/µl of 3- (4, 5-dim ethylthiazol-2-yl) -2, 5- diphenyl- 2H - tetrazolium bromide (MTT) (Sigma) was added to each well. After incubation for 4 h, the supernatant was replaced with 150 µl of dimethyl sulfoxide (Sigma). The absorption (A) was read at 570 nm using a spectrophotometer. The relative rate of cell proliferation was calculated as follows: (A-B)/(C+D) ×100%. (A) experiment group: Panc-1 CSCs+lymphocyte+PHA/OKT3; (B) positive control: lymphocyte+PHA/OKT3; (C) blank control: Panc-1 CSCs+PHA/OKT3. The experiments were performed in triplicate.

### Activating effect of Panc-1 CSCs modified DC on lymphocyte proliferation

To investigate the capacity of Panc-1 CSCs modified DC to activate the proliferation of lymphocyte, different groups of DC (Panc-1 CSC-DC, DC) were harvested as stimulator cells. After pretreatment with mitomycin C (25 µg/mL, Roche), the stimulator cells were co-cultured with 1×10^6^/ml allogeneic non-adherent lymphocyte (reactors) in 96-well plates at ratios ranging from 1∶10 to 1∶20 and were culture at 37°C, 5% CO_2_ for 96 h. The lymphocytes cultured alone without stimulating were set as controls. Then 20 µl 5 µg/µl of 3- (4, 5-dim ethylthiazol-2-yl) -2, 5- diphenyl- 2H – tetrazolium bromide (MTT) (Sigma) was added to each well. After incubation for 4 h, the supernatants were replaced with 150 µl of dimethyl sulfoxide (Sigma). The absorption (A) was read at 570 nm using a spectrophotometer. The relative number of lymphocyte after activation was calculated as: A experiment/A control×100%. The rate of cell proliferation was calculated as: (A experiment-A control)/A control×100%. The experiments were performed in triplicates.

### Cytokine detection by Elisa assay

To determine the stimulating capacity of dendritic cells on lymphocyte activation, the supernatants of the lymphocyte were collected 72 h after being stimulated with DC, and the concentrations of IFN-γ, IL-2, IL-10 were measured by ELISA kit from R&D according to the manufacture's guideline. The results were obtained by using a microplate spectrophotometer. The experiments were performed in triplicates.

### Cytotoxicity assay

The Panc-1 CSCs lysates modified DC and the primitive dendritic cells (1×10^5^/ml) were cultured at a ratio of 1∶10 with lymphocyte for 5 days. The non-adherent cells were collected and counted as effectors cells. The pancreatic cancer cells were plated in 12-well plate at a density of 1×10^5^/ml each. Effectors cells were co-cultured with pancreatic cancer cells at different ratio for 20 h in 37°C, 5% CO_2_ incubator. The cell-free supernatant was collected and analyzed by a non-radioactive LDH release kit according to the manufacturer's instructions. The LDH activity of effectors cells only was detected as controls, and the LDH activities in different reaction groups relative to controls group were calculated as the cytotoxicity of different DCs. The experiments were performed in triplicates.

### Statistical Analysis

The data were expressed as means±SD. Statistical differences between different groups were evaluated using the Student's t test. All analyses were performed using SPSS 18.0 software. P<0.05 was considered significant.

## Results

### Pancreatic CSCs culture and sphere-forming

**Figure 1:**
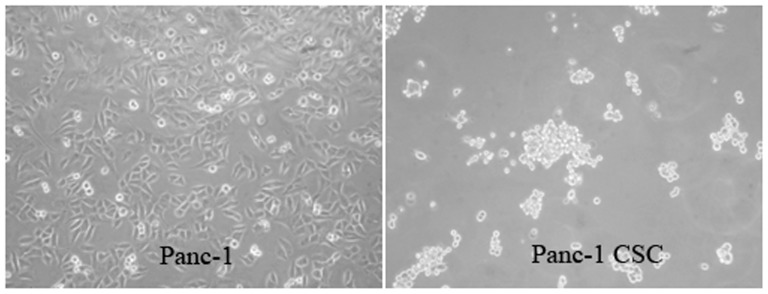
Panc-1 CSCs can be enriched under sphere forming conditions.

The sphere-forming abilities of cancer cells in serum free medium have long been used to enrich the stem-like cells. The relationship between the sphere forming abilities in serum free medium and the stem cell property of Panc-1 pancreatic cancer cells have been tested in our previously studies [9], [10]. Our preliminary studies found that Panc-1 cells can propagate to form spheres with stem cell properties. Moreover, these stem-like cells showed increased resistance to chemotherapy and increased migration ability. In this study, we collected the Panc-1 CSCs cultured under serum-free medium for further study of the immunological killing strategy (**Fig. 1**).

### The immunologic phenotype of Panc-1 sphere cells

**Figure 2:**
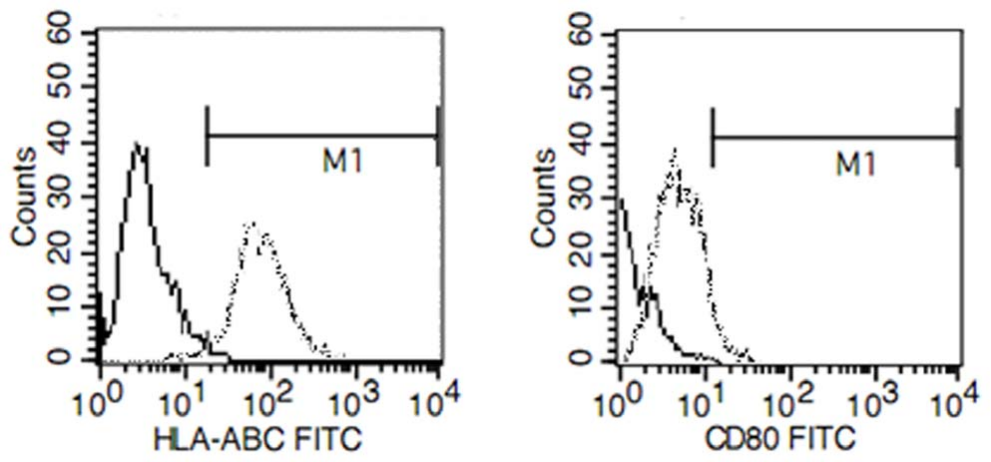
Flow cytometry analysis of HLA-ABC and CD80 expression on Panc-1 and Panc 1-sphere. Representative example of FCM analysis showed that MHC I and CD80 were lowly expressed in Panc-1 CSCs compared with Panc-1 cells. No significant differences were found for other immune molecules including CD86, HLA-DR, HLA-DQ, CD86, and CD1a. The expression of CD80 and HLA-ABC on Panc-1 CSCs are shown by a solid line. The expression of CD80 and HLA-ABC in Panc-1 cells are shown by a dotted line

We detected the expression of immunologic molecules on Panc-1 sphere by FACS methods. As shown in **Fig. 2**, Panc-1 sphere expressed low levels of HLA-ABC and CD80 compared with Panc-1 cells. We didn't find any differences between Panc-1 and Panc-1 spheres regarding other immune molecular markers including HLA-DR, HLA-DQ, CD86, and CD1a. The low expression of immune molecular markers on Panc-1 sphere indicates that the low stimulating ability of CSCs on immune system.

### Effect of pancreatic cancer cells on the proliferation of lymphocyte

**Figure 3:**
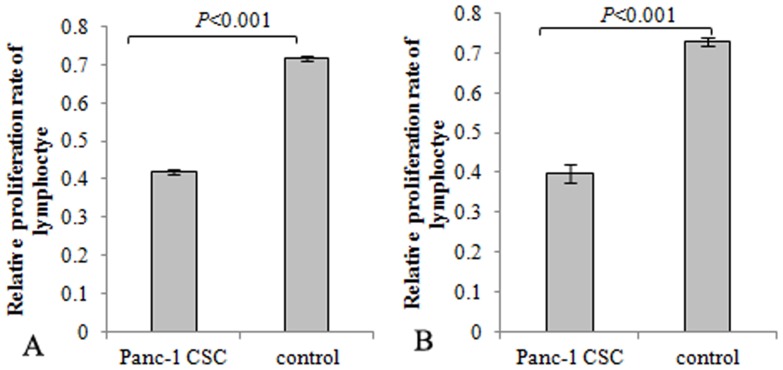
Panc-1 CSCs inhibited lymphocyte proliferation. Lymphocytes were stimulated to proliferate with PHA or anti-CD3 monoclonal antibodies. Panc-1 CSCs were co-cultured with lymphocyte at a ratio of 1∶10 in the presence of PHA or anti-CD3 monoclonal antibodies. Lymphocytes stimulated with PHA or anti-CD3 monoclonal antibodies solely were set as control. MTT assay indicated that lymphocyte proliferation promoted by PHA or anti-CD3 monoclonal antibodies was inhibited in the presence of Panc-1 CSCs.

CSCs have been reported to modulate immune and induce immune suppression through multiple mechanisms. To determine if pancreatic CSCs modulate the activation of lymphocytes, effects of Panc-1 CSCs on lymphocyte proliferation promoted by PHA or anti-CD3 monoclonal antibodies were assayed by MTT assay. Panc-1 spheres were co-cultured with lymphocyte. PHA or anti-CD3 monoclonal antibodies were used to activate the lymphocyte proliferation. The results showed that the proliferation of lymphocyte activated by PHA or anti-CD3 monoclonal antibodies was inhibited in the presence of pancreatic CSCs compared with controls (**Fig. 3**).

### The stimulating ability of CSCs lysates modified DC on lymphocyte proliferation

**Figure 4:**
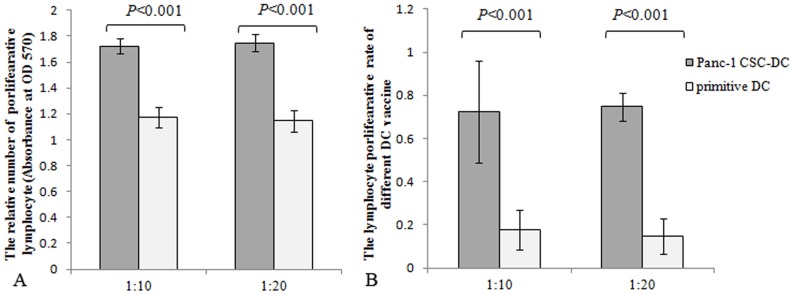
Panc-1 CSCs lysates modified DCs stimulated proliferation of lymphocyte. Different DCs (Panc-1 CSC DC, DC) were cocultured with lymphocyte (reactors) in 96-well plates at different ratios (1∶10, 1∶20). The lymphocytes cultured alone were set as controls. After 96 h, the relative cell number was calculated as absorbance by MTT methods. The experiments were performed in triplicate. A. The relative number of lymphocyte after stimulation can be reflected by absorbance. Panc-1 CSCs modified DC stimulated stronger proliferation of lymphocyte compared with DC groups. B. The activating effects of DCs on lymphocyte can be reflected by lymphocyte proliferation rate. The Panc-1 CSCs lysates modified DC achieved a significant high lymphocyte proliferation rate compared with DC groups.

To determine the effect of CSCs lysates modified-DC on the proliferation of lymphocytes, different proportion of DC (Panc-1 CSC DC and primitive DC) were co-cultured with lymphocytes. The activating effects of different DC on the proliferation of lymphocytes were demonstrated by MTT method. The proliferation of lymphocytes was demonstrated with the observance at OD570 nm. As shown in **Fig. 4**, DC loaded with Pancreatic CSCs lysates elicited strong proliferation of lymphocyte, (**Fig. 4A**). The activating effect of different DC on lymphocytes can be reflected by lymphocytes proliferation rate, and the activating effect of Panc-1 sphere lysates loaded DC on lymphocytes reached 72.4% and 74.7% at the ratio of 1∶10 and 1∶20, whereas the lymphocyte proliferation activated by DC only reached 17.5% and 14.6% (**Fig. 4B**).

### Pancreatic CSCs lysates modified DC provoked secretion of IFN-γ IL-2 and IL-10 on lymphocytes

**Figure 5:**
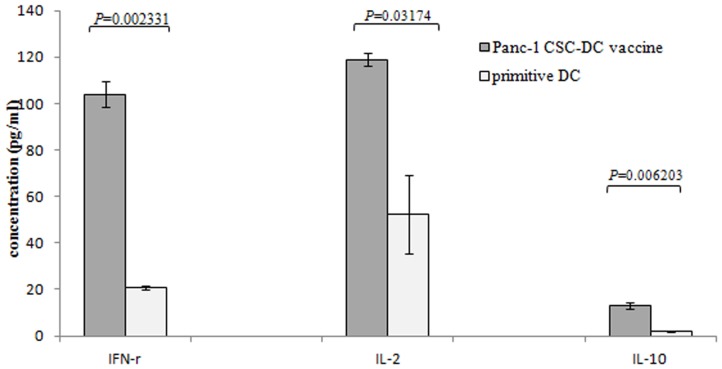
Pancreatic CSCs lysates modified DCs provoked the secretion of IFN-γ, IL-2 and IL-10 on lymphocyte. Different DCs were co-cultured with 1×10^6^/ml lymphocyte in 96-well plates. The supernatants of the lymphocyte were collected 72 h after being stimulated with DC, and the concentrations of IFN-γ, IL-2, IL-10 were measured by ELISA assay. Pancreatic CSCs lysates modified DC provoked significant secretion of IFN-γ, IL-2 and IL-10 on lymphocyte.

To determine the activation of lymphocytes by Pancreatic CSCs modified DC, we detected the cytokines secreted by T-cells according to materials and methods. DC pulsed with pancreatic cancer cell lysates induced secretion of Th1 cytokine IFN-γ, IL-2 and the Th2 cytokine IL-10. Our results showed that the up regulation of IFN-γ was especially significant for the Panc-1 sphere modified DC, and the secretion of IFN-γ increased to 5.03 fold compared to primitive DC group. The secretion of IL-2 increased to 2.28 fold compared to primitive DC group (**Fig. 5**). Meanwhile, the Th2-associated cytokine IL-10 also increased in Panc-1 CSCs antigen loaded DC. The amount of IL-10 in the supernatant increased up to 7.3 fold in Panc-1 sphere lysates modified DC relative to the primitive DC, but significantly lower than that of the Th1 associated cytokine (**Fig. 5**).

### Pancreatic CSCs modified DC induced strong killing effect on pancreatic CSCs

**Figure 6:**
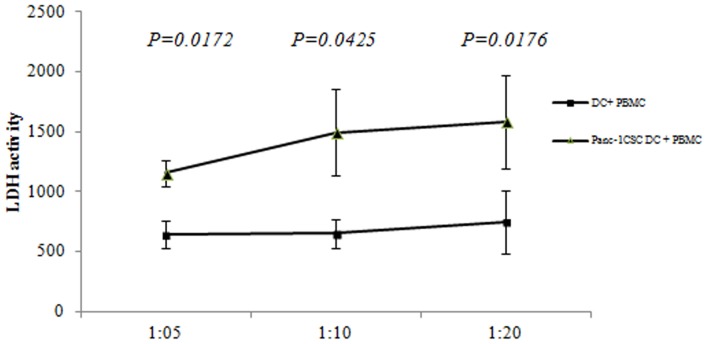
Pancreatic CSCs lysates modified DCs induced significant killing effect on pancreatic CSCs. Different DCs were co-cultured at a ratio of 1∶10 with lymphocyte for 5 days. The non-adherent cells were collected and counted as effectors cells. Effectors (1×10^6^ cells) were cocultured with the pancreatic cancer cells at different ratio for 20 h at 37°C in a 5% CO_2_ incubator. The cell-free supernatant was collected and analyzed by LDH assay. The Panc-1 CSCs lysates modified DC showed stronger killing effect on Panc-1 CSCs compared with controls.

**Figure 7:**
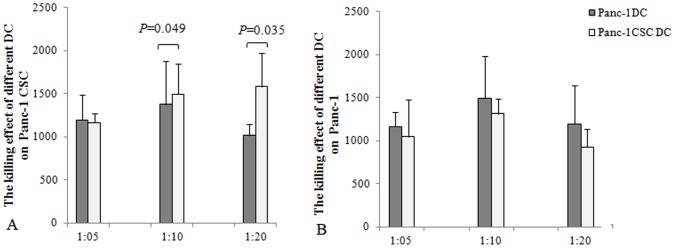
The killing effect of different DCs on Panc-1 and Panc-1 CSCs. Different DCs (Panc-1DC, Panc-1CSC DC) were co-cultured at a ratio of 1∶10 with lymphocyte for 5 days. The non-adherent cells were collected and counted as effectors cells. Effectors (1×10^6^ cells) were cocultured with the pancreatic cancer cells (Panc-1, Panc-1 CSCs) at different ratio for 20 h at 37°C in a 5% CO2 incubator. The cell-free supernatant was collected and analyzed by LDH assay. A. The Panc-1 CSCs lysates modified DC showed stronger killing effects on Panc-1 CSCs compared with Panc-1 lysates modified DC. B. Panc-1 CSCs lysates modified DC had comparable killing effects on Panc-1 cells compared with Panc-1 DC vaccine.

We further compared the specific killing effect of lymphocyte activated by pancreatic CSCs modified DC. Autologous lymphocyte stimulated with CSC-DC were collected and co-cultured with Panc-1 CSCs at different ratio. The specific killing effect was detected by LDH methods. The lymphocytes activated by DC loaded with Panc-1 CSCs debris showed significant killing effect on Panc-1 CSCs compared to DC controls at different ratio (**Fig. 6**). Moreover, we found that primitive Panc-1 cell lysates loaded DC provoked a weaker killing effect on Panc-1 CSCs compared to Panc-1 CSCs lysates modified DC (**Fig. 7A**). However, Panc-1 CSCs modified DC provoked comparable killing effects both on Panc-1 CSCs and Panc-1 cells. Though the killing effects were weaker on Panc-1 cell compared with Panc-1 DC, there was no significant difference between the Panc-1 DC and Panc-1 CSC- DC (**Fig. 7B**). This killing effect was especially significant at a lower proportion of 1∶10 and 1∶20 with lymphocytes.

## Discussion

CSCs have been notorious for its resistance to therapy and may be the cause for relapse of malignant tumors, as previously reported [10]–[11]. Development of antitumor immunity may thus be an effective alternative approach to eradicate pancreatic CSCs. Unfortunately, CSCs can breach the body's immune surveillance through multiple mechanisms. CSCs have been reported to be weak immunogenic. Ohlfest et al reported that human CD133+ gliomas cells express low levels of MHC I or natural killer (NK) cell activating ligands. Low expression of these immune stimulating molecules can help the gliomas CSCs to evade the adaptive and innate immune attacks [12]. Consistently in our study, flow cytometry analysis revealed that pancreatic CSCs express low levels of HLA-ABC and CD80. Low expression of MHC molecules may weaken the identification of CSCs antigen by human body's immune system. Low expression of CD80 may lead to immune anergy [13]. The observed immune characteristics suggested low immune stimulating abilities of pancreatic CSCs to the immune system. CSCs were also reported to modulate immune responses. Heimberger et al reported that CSCs contributes immune evasion through inducing regulatory T cells and secreting specific immunosuppressive molecules [14], [15]. It was also reported that the malignant melanoma initiating cells can modulate the antitumor immune response through inhibiting T-cell activation and inducing Treg cells [14]. Our study found that the activation of lymphocyte stimulated by PHA or anti-CD3 monoclonal antibodies was inhibited in the presence of Panc-1 sphere. This indicated that pancreatic CSCs may have immune suppressive characteristics. Breaking the immune tolerance of pancreatic CSCs and induction of immune responses against CSCs may be effective and promising strategies to eliminate CSCs.

DCs are professional antigen-presenting cells that play a key role in inducing and driving of primary immune responses, rendering them as an essential target in generating therapeutic immunity against cancers [16]. Modification of DCs with tumor antigen can induce T-cell activation and antitumor immune response. DC-based vaccination represents such a promising and powerful strategy for eliciting antitumor immunity for cancer treatment. Generation of DC loaded with CSCs associated antigens may present a novel and valuable method for eliciting the immune response to eradicate CSCs. It had been reported that cytotoxic T lymphocyte against CSCs can be induced by fusion of CSCs with DC or transfection of CSCs mRNA into dendritic cells [17]–[18]. In this study, we charged DC with pancreatic CSCs debris. After co-culture with lymphocytes, this modified DC activates and stimulates lymphocyte proliferation and secretion of INF-γand IL-2. The up-regulation of IFN-γ, which is a strong antitumor cytokine, was especially significant. Meanwhile, we found that the Th2 associated cytokine IL-10 also increased after DC stimulation. IL-10 is a potent immunosuppressive cytokine which inhibits T cell activation. Though up-regulation of IL-10 was much lower compared with the Th1 associated cytokine, inhibition of IL-10 may enhance the immune response against CSCs.

Our further study revealed that lymphocyte activated by Panc-1 CSCs lysates modified DC showed significant killing effects both on Panc-1 and Panc-1 CSCs. Moreover, the killing effect on Panc-1 CSCs provoked by Panc-1 CSC-DC was stronger, compared with Panc-1 DC. Considering that pancreatic cancer is resistant to nearly all currently available therapies, and the therapeutic resistance may be attributed to CSCs. Development of immunotherapy to pancreatic CSCs becomes a promising approach to treat pancreatic cancer in the near future.

To date, isolation of pancreatic CSCs has been achieved mainly through specific surface marker-dependent cell selection. Reported cell surface markers include CD44, CD24, CD133, ESA, and others for pancreatic CSCs [2], [19]. However, pancreatic CSCs selected through those cell surface markers may be heterogeneous, as none of these markers appear to selectively characterize a pure population of CSCs [20]–[21]. On the other hand, cultivation of CSCs spheres in stem cell conditioned culture system may be another approach to enrich pancreatic CSCs. In current study, we enriched pancreatic CSCs by using nonadherent sphere culture system. The debris of the cell sphere was used to charge DC and collect CSCs modified DC. The immune reaction induced by such an approach may directly target the majority of pancreatic CSCs population.

In summary, our results showed that DC-based immune therapy can be an ideal strategy to eliminate pancreatic CSCs. This promising immune treatment worth further development considering the intrinsic resistance of pancreatic CSCs to existing therapies.

The authors declare that there is no conflict of interests regarding the publication of this paper.
